# Artemisinin attenuated ischemic stroke induced cell apoptosis through activation of ERK1/2/CREB/BCL-2 signaling pathway *in vitro* and *in vivo*

**DOI:** 10.7150/ijbs.69892

**Published:** 2022-07-11

**Authors:** Tangming Peng, Shuai Li, Linlin Liu, Chao Yang, Mohd Farhan, Ligang Chen, Qiaozhu Su, Wenhua Zheng

**Affiliations:** 1 Faculty of Health Science, University of Macau, Taipa, Macau, China; 2 Department of Neurosurgery, Affiliated Hospital of Southwest Medical University and Neurosurgical Clinical Research Center of Sichuan Province, Luzhou, China; 3 Institute for Global Food Security, School of Biological Sciences, Queen's University Belfast, Belfast BT9 5DL, United Kingdom

**Keywords:** Ischemic stroke, artemisinin, neuronal damage, ERK1/2/CREB/BCL-2

## Abstract

Ischemic stroke is characterized by the presence of both brain ischemic and reperfusion-induced injuries in the brain, leading to neuronal dysfunction and death. Artemisinin, an FDA-approved antimalarial drug, has been reported to have neuroprotective properties. However, the effect of artemisinin on ischemic stroke is not known. In the present study, we investigated the effect of artemisinin on ischemic stroke using an oxygen-glucose deprivation/reperfusion (OGD/RP) cellular model and a mouse middle cerebral artery occlusion (MCAO) animal model and examined the underlying mechanisms. The obtained results revealed that a subclinical antimalarial concentration of artemisinin increased cell viability and decreased LDH release and cell apoptosis. Artemisinin also attenuated the production of reactive oxygen species (ROS) and the loss of mitochondrial membrane potential (Δψm). Importantly, artemisinin attenuated the infarction volume and the brain water content in the MCAO animal model. Artemisinin also improved neurological and behavioural outcomes and restored grasp strength and the recovery of motor function in MCAO animals. Furthermore, artemisinin treatment significantly inhibited the molecular indices of apoptosis, oxidative stress and neuroinflammation and activated the ERK1/2/CREB/BCL-2 signaling pathway. Further validation of the involved signaling pathway by the ERK1/2 inhibitor PD98059 revealed that inhibiting the ERK1/2 signaling pathway or silencing ERK1/2 reversed the neuroprotective effects of artemisinin. These results indicate that artemisinin provides neuroprotection against ischemic stroke via the ERK1/2/CREB/BCL-2 signaling pathway. Our study suggests that artemisinin may play an important role in the prevention and treatment of stroke.

## 1. Introduction

Artemisinin (ART) is a traditional Chinese medicine that has been used as an anti-malaria drug in the clinic for decades. In addition to its antimalarial effects, artemisinin and its derivatives have also been reported to have anti-cancer, anti-allergic and anti-inflammatory effects that are mediated by a large number of molecular targets 1-3. Moreover, we recently reported that artemisinin and its derivatives have neuroprotective effects and may have potential applications in the prevention and treatment of brain disorders 4-8. However, the protective effects and potential mechanisms by which artemisinin protects against ischemic stroke-induced cell injury have not yet been reported and need to be further investigated.

Stroke is the second leading cause of death worldwide, killing approximately 6 700 000 individuals each year 9, 10. Ischemic stroke accounts for more than 80% of cases and is mainly due to an abrupt blockage of the internal carotid or the middle cerebral arteries 11, 12. It has been reported that the five-year recurrence risk of ischemic stroke ranges from 15% to 40%, and according to Wolfe's survey^13^, and the number of ischemic stroke patients will increase approximately 30% in 2023 compared to 1983, urging the development of more effective therapies. Ischemic stroke, which results from an acute cerebral artery block, can be caused by proximal middle cerebral artery occlusion (MCAO) (large vessel occlusion), distal MCAO (small vessel occlusion), or by thrombotic occlusion 14. Thus, the MCAO experimental model, which mimics the blockage of blood flow in patients, is the most commonly used transient focal cerebral ischemic and reperfusion injury animal model, as it can be used to study the pathophysiology of ischemic stroke and its underlying mechanisms in preclinical studies 15-17.

Ischemic stroke is characterized by the presence of both ischemic and reperfusion-associated brain injuries, leading to neuronal dysfunction and death. During ischemic stroke, the initial injury occurs within a few minutes and prevents the blood supply to the focal core of the ischemic injury area 18, resulting in a significant lack of oxygen and glucose to neurons. At the blockage region, which is the focal core of the ischemic injury area, many cells, especially neurons, die via cell necrosis, making the attempt to restore the blood and oxygen supply almost worthless 19. During ischemic and reperfusion-induced injuries, due to anaerobic metabolism and lactic acid accumulation, ATP levels decline, leading to ATP-dependent ion transport dysfunction, calcium overload in mitochondria, cells swelling, and cell death via different mechanisms, including necrosis, apoptosis, and autophagy^20^. Therefore, many molecular and cellular signaling pathways are activated 21. Cell apoptosis is a critical mechanism in ischemic stroke, and inhibiting apoptosis may attenuate ischemic and reperfusion induced bran injuries that follow ischemic stroke 22. These findings further indicate that apoptosis may contribute to identifying the most effective targets for ischemic stroke treatment, offering new hope for patients suffering from this serious disease.

Cells have extremely complex activities that need to be strictly regulated. During evolutionary development and natural selection, a complex signal transduction network was gradually established, which is formed by different signal transmission pathways through mutual connections and actions 23, 24. The mitogen-activated protein kinase (MAPK) signaling pathway plays a pivotal role in controlling different physiological processes such as cell growth, development, division and death 25. Extracellular signal-regulated kinase (ERK) belongs to the MAPK family, and this signaling pathway is involved in the regulation of cell growth, development and division 26. Researchers have shown that the phosphorylation of ERK in the cerebral ischemia-reperfusion side of mice was significantly reduced 27, 28, indicating its role in the ischemic and reperfusion induced injury. Our study showed that ERK was significantly activated after the administration of artemisinin. However, the effect of artemisinin on ischemic stroke is not known. Thus, we assessed the protective effect of artemisinin against ischemic stroke using an OGD/RP cellular model and an MCAO animal model. The results showed that artemisinin significantly protected neuronal cells and brain tissues from ischemic stroke via the ERK1/2/CREB/BCL-2 signaling pathway. Our findings suggest the potential use of artemisinin in the treatment of ischemic stroke.

## 2. Materials and Methods

### 2.1 Materials

Artemisinin (ART) (purity≥98%) was purchased from Dalian Meilun Biotechnology, China. Glucose-free and glucose-containing Dulbecco's modified Eagle's medium (DMEM) was obtained from GIBCO^TM^, Invitrogen Corporation. Bovine serum albumin (BSA) and fetal bovine serum (FBS) were obtained from GIBCO^TM^. Invitrogen Corporation. Lipofectamine 2000 and 2,3,5-triphenyltetrazolium chloride (TTC, Cat# T8877) were obtained from Sigma Aldrich (St. Louis, MO, USA). MTT and Hoechst 33342 were purchased from Molecular Probes (Eugene, OR, USA). An Annexin V-FITC/PI apoptosis detection kit was purchased from BD Biosciences (San Diego, CA, USA). The LDH assay kit, Caspase 3 assay kit, DCFH-DA reagent, JC-1 dye, Nissl staining solution, TUNEL assay kit, MDA assay kit and SOD assay kit were obtained from Beyotime Institute of Biotechnology (Shanghai, China). Monofilament nylon sutures were purchased from Beijing Sunbio Biotech Co. Ltd. (Beijing, China). Anti-phospho-ERK1/2, anti-ERK1/2, anti-phospho-CREB, anti-NeuN, anti-GFAP, IL-18, IL-1β, NF-κB and anti-GAPDH antibodies were purchased from Cell Signaling Technology (Woburn, MA, USA). Anti-BAX and anti-BCL-2 antibodies were purchased from Signalway Antibody (College Park, Maryland, USA). The biotinylated anti-rabbit IgG and TRITC-conjugated anti-rabbit IgG secondary antibodies were purchased from Cell Signaling Technology (Woburn, USA). The ERK1/2 inhibitor PD98059 was ordered from Calbiochem (San Diego, CA, USA). siERK1/2 was purchased from Genepharma (Shanghai, China).

### 2.2 Cell Culture

PC12 cells were purchased from the Cell Bank of Sun Yat-sen University (Guangzhou, China). The cells were grown in DMEM with 10% fetal bovine serum (FBS), 1% streptomycin and 1% penicillin, and maintained at 37°C in a humidified atmosphere of 5% CO_2_.

Primary cultured neurons from C57BL/6J embryonic (E18) foetuses were cultured for 6-7 days in DMEM/F12 supplemented with 2% B27, 20 μM L-glutamine, 15 mM HEPES, 10 U/ml penicillin, and 10 μg/ml streptomycin.

### 2.3 Animals

The animal experimental protocol was approved by the Animal Ethics and Welfare Committee of the University of Macau and was carried out in accordance with approved guidelines and regulations. Male C57BL/6J mice were used and the body weights of the mice was 21-23 g. The mice were obtained from the Animal Research Core in the Faculty of Health Science of the University of Macau and kept under controlled temperature (24-26 °C), humidity and lighting (12 hours light/dark cycle). Food and water were available *ad libitum* throughout the experiment.

### 2.4 Establishment of the OGD/RP model

PC12 cells and primary cultured neurons were cultured in glucose-containing DMEM with all the standard supplements for 24 hours and then placed in a special incubator loaded with a gas mixture containing 5% CO_2_, 1% O_2_ and 94% N_2_ for 2, 4, 6, 8 and 24 hours at 37 °C before reperfusion. Cells were then incubated in glucose-containing DMEM supplemented with 10% FBS at 37 ℃ in an O_2_-free incubator under humid conditions for another 22, 20, 18, 16 and 0 hours. Then, the cells were incubated with MTT (0.5 mg/mL) for 3 hours in the dark at 37 ºC. The medium was replaced with DMSO (100 μL/well). The absorbance at 570 nm was measured on a BIO-RAD680 microplate reader (Thermo Fisher, MA, USA).

### 2.5 Assessment of cell viability using MTT assay

PC12 cells and primary cultured neurons were seeded in 96-well plates (5×10^3^ cells per well for PC12 cells and 7x10^3^ cells per well for primary cultured neurons) in DMEM with 0.5% FBS. After 24 hours, the cells were pretreated with different concentrations of artemisinin (6.25-50 μM) or 0.1% DMSO for 2 hours and then subjected to 4 hours of OGD followed by 20 hours of reperfusion. Cell viability was tested by MTT assays. After 24 hours, PC12 cells and primary cultured neurons were incubated with MTT (0.5 mg/mL) for 3 hours in the dark at 37ºC. Then, the medium was replaced with DMSO (100 μL/well). The absorbance at 570 nm was measured on a BIO-RAD 680 microplate reader (Thermo Fisher, MA, USA).

### 2.6 Assessment of cell cytotoxicity using LDH assays

Cell cytotoxicity induced by OGD/RP model was assessed by measuring LDH activity as previously described 29. Briefly, PC12 cells and primary cultured neurons were seeded in 96 well plates (1×10^4^ cells/well). After being pretreated with different concentrations of artemisinin or 0.1% DMSO for 2 hours, PC12 cells and primary cultured neurons were incubated with or without OGD/RP for 4 hours followed by 20 hours of reperfusion. LDH release into the medium was measured according to the LDH assay kit instructions, and the results were recorded on an Infinite M200 PRO multimode microplate at a wavelength of 490 nm.

### 2.7 Measurement of cell apoptosis using Hoechst 33342 staining

A Hoechst 33342 staining assay was used to study the effect of artemisinin on apoptosis as previously described with minor modifications 30. After being pretreated with artemisinin or 0.1% DMSO for 2 hours, PC12 cells and primary cultured neurons were incubated with or without OGD/RP for 4 hours followed by 20 hours of reperfusion. Then, PC12 cells and primary cultured neurons were rinsed with PBS followed by and fixed using 4% cold paraformaldehyde (PFA) for 15 minutes. The fixed cells were then rinsed and stained with Hoechst 33342 dye for 3-5 minutes at room temperature and visualized by an EVOS FL Imaging System (Thermo Fisher Scientific, Waltham, MA, USA). The percentage of apoptotic cells was calculated and analyzed.

### 2.8 Measurement of cell apoptosis using the Annexin V-FITC/PI assay

Cellular apoptosis was further analyzed using FACS according to the Annexin V-FITC/PI Kit instructions. Briefly, PC12 cells were seeded into 6-well plates (2×10^5^ cells/mL). After being treated as indicated, the cells were harvested and washed twice with cold PBS and suspended in the binding buffer. Then, PC12 cells were incubated with Annexin V-FITC (10 μg/mL, 5 μL) and 10 μL of PI (20 μg/mL). The number of apoptotic cells was calculated by flow cytometry (BD FACS Calibur).

### 2.9 Measurement of cell apoptosis using a caspase 3 activity assay

Measurement of caspase 3 activity was performed using a caspase 3 activity assay kit according to the manufacturer's protocol. Briefly, after the indicated treatments, PC12 cells and primary cultured neurons were lysed and centrifuged for 5 min. The collected cells were resuspended in the lysis buffer for another 15 min before being centrifuged for 15 min. The supernatant was collected and incubated (50 μL) with 40 μL of assay buffer and 10 μL of the Caspase 3 substrate Ac-DEVD-pNA for 2 hours at 37 °C. Then, caspase 3 activity was measured at 405 nm using BIO-RAD680 microplate reader (Thermo Fisher, MA, USA).

### 2.10 Measurement of intracellular ROS

Intracellular ROS levels were evaluated using DCFH-DA. After the indicated treatments, the medium was replaced with 5 μM DCFH-DA in DMEM for 1 hour away from the light. Then, PC12 cells and primary cultured neurons were rinsed twice with PBS and photographed by an EVOS FL Imaging System (excitation wavelength: 488 nm; emission wavelength: 525 nm). Intracellular ROS levels were quantified using Image J software. All % ROS levels were calculated compared to the ctrl group.

### 2.11 Measurement of mitochondrial membrane potential (△ψm)

The JC-1 assay was used to measure the Δψm. In short, PC12 cells and primary cultured neurons were seeded into 96-well plates, and after being treated, the cells were incubated with JC-1 dye. Images were taken by an EVOS FL Imaging System. The ratio of JC-1 red/green fluorescence intensity was calculated by Image J software, and the value was calculated relative to the control group.

### 2.12 Western blotting

Western blotting was performed as previously described 31. In brief, after the appropriate treatments, the cells were collected and lysed with RIPA buffer, and the protein concentration was determined using a BCA kit. Equal amounts of protein were separated using polyacrylamide gel electrophoresis (PAGE) and transferred onto polyvinylidene fluoride (PVDF) membranes. Then, the membranes were blocked with 5% nonfat milk in TBST for 1-2 h at room temperature. Primary antibodies against P-ERK1/2, T-ERK1/2, P-CREB, BAX, BCL2, IL-18, IL-1β, NK-κB, GAPDH and Actin were used to detect the different proteins after an overnight incubation at 4 °C overnight. The membranes were then incubated with HRP-conjugated secondary antibodies after being washed with TBST. The protein bands were visualized using Clarity Western ECL substrate with a Bio-Rad Gel Doc XR documentation system. Blot intensity was analyzed using image J analysis software.

### 2.13 ERK1/2 silencing using siRNA in PC12 cells

Interfering RNA (siRNA) targeting ERK1 and ERK2 (ERK1/2) and scrambled control siRNA were synthesized by Genepharma. Cells were transfected with the target siRNA or scrambled siRNA using Lipofectamine 2000 for 6 hours and were then place in normal medium. The efficiency of ERK1/2 knockdown was assessed by western blotting using specific antibodies, and cell viability was determined by MTT assays.

### 2.14 Establishment of the middle cerebral artery occlusion (MCAO) model

The mice were randomly divided into the control group (Ctrl), MCAO model group (Model) and model plus artemisinin (2, 6, 18 mg/kg, i.p.) groups (ART), and 20 mice were used in each group. To establish the MCAO mouse model, the animals were anaesthetized with an intraperitoneal injection (i.p.) of 1% sodium pentobarbital, and the right common carotid artery (CCA), the external carotid artery (ECA) and the internal carotid artery (ICA) were exposed. The ICA was temporarily blocked using a microarterial clip, and a surgical nylon filament was inserted from the ECA to the ICA to block blood flow. Then, the filament was withdrawn, and the ECA was ligated. Cerebral blood flow (CBF) in the middle cerebral artery (MCA) was measured by a Laser Specke Perfusion Imager System (RWD, Shenzhen, China) through the skull (Fig. S1). All the animals with a CBF reduction of < 80% in MCA regional center were excluded from the study. After 1 hour of occlusion, reperfusion was allowed by gently removing of the monofilament. Body temperature was maintained at 37 °C using a heat lamp during the surgery. Artemisinin was administered via intraperitoneal injection.

### 2.15 Infarct volume and brain water content measurements

The mice were sacrificed by decapitation 24 hours after the surgery and the brains were immediately collected. Coronal brain slices (2 mm thickness) were stained with 2,3,5-triphenyltetrazolium chloride (TTC). In short, after the animals were sacrificed, the brains were removed quickly, and washed with cold PBS. Then, the brains were chilled at -20 ℃ for 30 min, and cut into coronal brain slices with a thickness of 2 mm. The brain slices were immersed in 2% TTC solution at 37 ℃ for 30 min in the dark. To fully stain the slices, the container was shaken slightly every 5 min. Finally, the brain slices were removed from TTC solution and fixed with 4% PFA overnight. Brain sections from each group were photographed and the infarct size was calculated using the following formula: corrected infarct volume % = [contralateral hemisphere area - (infarct area in ipsilateral hemisphere/contralateral hemisphere area)] x 100%. The brain water content was measured using a standard wet/dry weighing method in which the collected brains were divided into ischemic and nonischemic hemispheres and weighed (wet weight). Afterwards, the samples were dried for 48 hours in an oven at 95 °C and the tissues were weighed again (dry weight). The brain water content was calculated using the following equation: brain water content = [(wet tissue weight - dry tissue weight)/wet tissue weight] × 100%.

### 2.16 Neurological evaluation and behaviour analyses

The neurological deficit score was obtained by an investigator who was blinded to the groups of the animals. The neurological score (Zea-Longa Scale) was used to evaluate neurological function at 0 and 24 hours after drug administration 32. The score criteria were as follows: 0 points, no symptoms of neurological damage; 1 point, slight neurological impairment and an inability to fully extend the contralateral forepaw; 2 points, severe neurological deficits, circling continuously to the contralateral side but standard posture at rest; 3 points, severe neurological deficits, falling to the injured side; and 4 points, no spontaneous autonomic activity, loss of consciousness. Grip strength test aimed to assess the recovery of the motor function of the affected limbs. All animals were evaluated 24 hours after drug administration. Three consecutive trials were carried out to measure the forelimb-strength of each animal. The strength of the affected limb was measured using a grip strength meter (Bioseb, USA). The pole-jump test was further performed after drug administration. The time that the mice stayed on the pole, fell down or jumped was recorded (when mice stayed on the pole for more than 60 seconds, 60 seconds was the time recorded, and if the mice fell down or jumped off after 120 seconds, 120 seconds was the time recorded).

### 2.17 Nissl Staining and TUNEL Assays

Neuronal survival was examined using Nissl staining and neuronal cell apoptosis was measured using the TUNEL assay as previously described 33. Nissl staining was performed according to the manufacturer's protocol. Briefly, samples were stained with Nissl staining solution and then dehydrated with ethanol and cleared with xylene. For the TUNEL assay, the samples were incubated with the TUNEL reaction mixture for 1 hour at 37 °C in the dark. Color development was performed in DAB solution. Images were taken using a microscope (EVOS FL Imaging System). Analyses were performed on 8 sections per sample, and there were 4 animals in each group.

### 2.18 Immunofluorescence (IF) and immunohistochemistry (IHC)

Brain slices were processed for immunofluorescence and immunohistochemistry as previously described 34. Mice were transcardially perfused with 0.1 M phosphate buffer followed by 4 % PFA in 0.1 M phosphate buffer. Coronal sections of the collected brains were examined (10 μm frozen sections for IF and 4 μm paraffin sections for IHC). For immunofluorescence analysis, samples were incubated with anti-NeuN primary antibodies (1:200) for 24 hours at 4 °C. After being washed 3 times with PBS, the slices were incubated with TRITC-conjugated anti-rabbit IgG (1:500) at room temperature for 1 hour. Images were acquired with a Nikon A1 confocal microscope. For immunohistochemical analysis, brain sections were dewaxed and hydrated before antigen retrieval, incubated with 3% H_2_O_2_ for 30 min and blocked with 10% bovine serum albumin (BSA). After that, the samples were incubated with the primary antibody (GFAP, 1:500) overnight at 4 °C. Afterwards, the samples were incubated with a biotinylated anti-rabbit IgG secondary antibodies (1:500) at room temperature for 1 hour and color development was performed with DAB solution. Images were taken using an EVOS FL Imaging System. Analyses were performed on 8 sections per sample, and there were 4 animals in each group.

### 2.19 Measurement of SOD and MDA activities

Superoxide dismutase (SOD) and malondialdehyde (MDA) activities were determined by a total Superoxide Dismutase Assay Kit with WST-8 and a Lipid Peroxidation MDA Assay Kit, according to the manufacture's protocols. Briefly, SOD activity was determined by measuring the production of a water-soluble formazan dye at a wavelength of 450 nm. MDA levels were determined by measuring the red products resulting from the reaction of MDA and thiobarbituric acid at a wavelength of 532 nm. Absorbances were measured using a BIO-RAD680 microplate reader (Thermo Fisher, MA, USA).

### 2.20 Statistical analysis

Statistical analysis was performed using GraphPad Prism 7.0 statistical software (GraphPad Software, Inc., San Diego, CA, USA). All values are presented as the mean ± SEM. Multiple comparisons were performed by one-way ANOVA using Tukey's multiple comparison post hoc-test and p<0.05 was considered statistically significant.

## 3. Results

### 3.1 Artemisinin attenuated OGD/RP-induced loss of cell viability and cytotoxicity in PC12 cells

**Figure 1:**
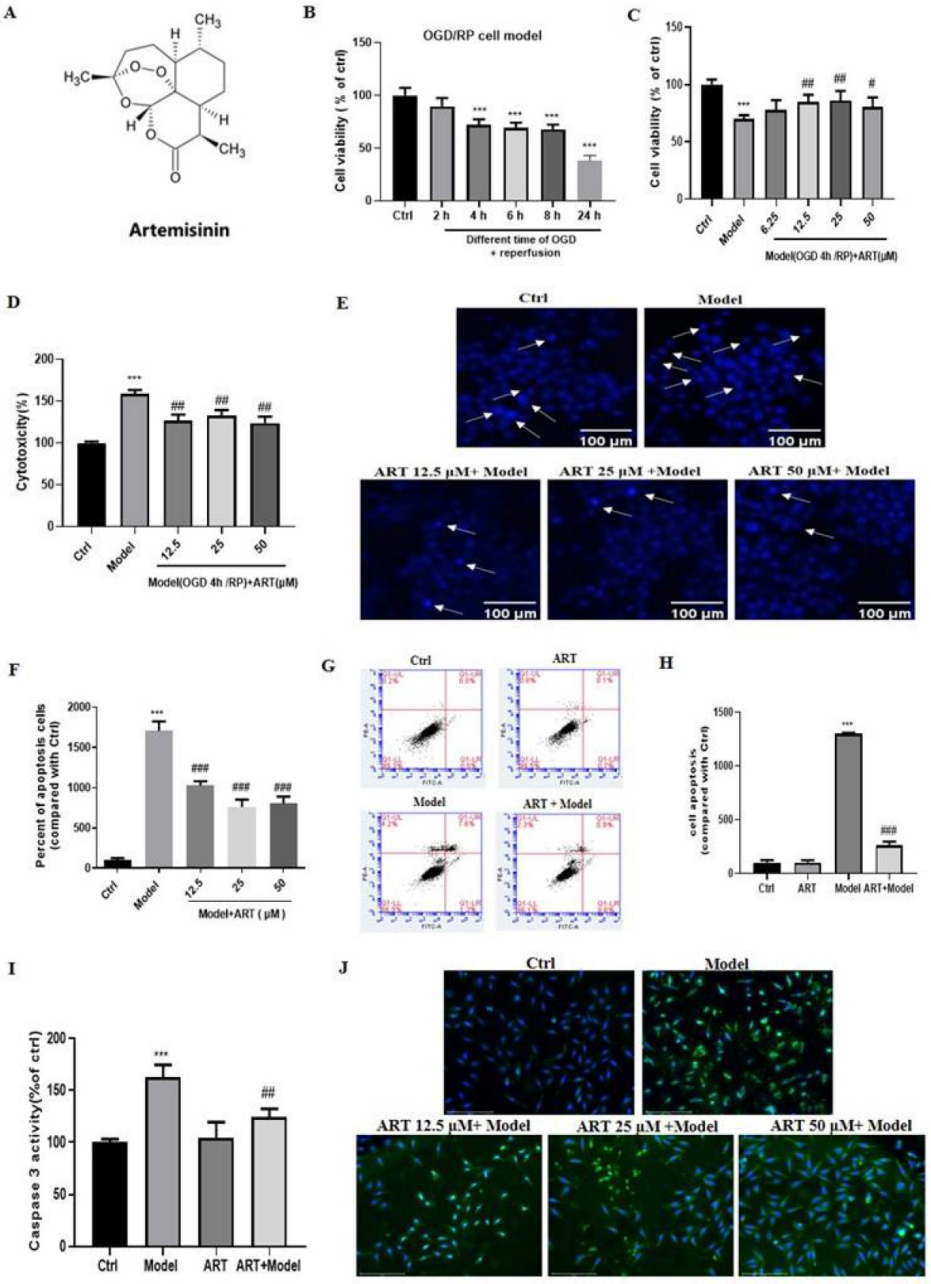
** Artemisinin attenuated OGD/RP-induced loss of cell viability and cytotoxicity in PC12 cells. (A)** Structure of artemisinin. **(B)** Cells were treated with different durations of OGD followed by reperfusion and cell viability was measured using MTT assay, ^***^ *p*< 0.001 were considered significantly different; **(C)** Cells were pretreated with different concentrations of artemisinin and then incubated with OGD for 4 hours followed by 20 hours of reperfusion and cell viability was measured using MTT assay. **(D)** Cells were pretreated with different concentrations of artemisinin and then incubated with OGD for 4 hours followed by 20 hours of reperfusion and the cytotoxicity was measured by lactate dehydrogenase (LDH) assay. The percentage was calculated by the ratio of the optical density (OD) values compared with ctrl group. **(E)** Cells were pretreated with artemisinin at the indicated concentrations and then subjected to OGD/RP conditions. Representative images of Hoechst 33342 staining. Scale bar: 100 μm. **(F)** Quantitative analysis of Hoechst 33342 staining. **(G)** Cells were pretreated with 25 μM artemisinin and then subjected to OGD/RP. Representative images of Annexin V-FITC/PI staining detected by flow cytometry. (H) Quantitative analysis of Annexin V-FITC/PI staining. (I) Cells pretreated with 25 μM artemisinin and then subjected to OGD/RP. The activity of caspase 3 was measured by caspase 3 activity assay. (J) Cells pretreated with 12.5 μM, 25 μM, 50 μM artemisinin and then subjected to OGD/RP. The expression level of caspase 3 (green) was detected by immunofluorescence. Scale bar: 150 μm. ^***^ *p* < 0.001, versus ctrl group; ^#^ *p* < 0.05, ^##^ *p* < 0.01, ^###^ *p* < 0.001, versus model group were considered significantly different. The assay was repeated at least 3 times.

To evaluate the cytotoxicity of OGD/RP, PC12 cells were subjected to different durations of OGD, which resulted in a time-dependent decrease in cell viability in a time-dependent manner (Fig. 1B). OGD for 4 hours caused a significant loss (approximately 30%) of viability; thus, this time was used in subsequent experiments to examine neuroprotection. To investigate the protective effect of artemisinin, PC12 cells were pretreated with artemisinin or 0.1% DMSO at concentrations ranging from 6.25 to 50 μM for 2 hours and then exposed to OGD for 4 hours and reperfusion for 20 hours, and viability was analyzed by MTT assays. The cell viability in the model group decreased significantly compared with that in the control group, and artemisinin reversed OGD/RP-induced death in a concentration-dependent manner. The protective effect of artemisinin started at a concentration of 6.25 μM, was significant at 12.5 μM, peaked at 25 μM and slightly decreased from at 50 μM (Fig. 1C). The LDH assay, results further revealed that artemisinin attenuated OGD/RP-induced LDH release (Fig. 1D).

### 3.2 Artemisinin attenuated OGD/RP-induced PC12 cell apoptosis

Hoechst 33342 staining was used to measure apoptosis by detecting apoptotic changes in nuclei. The results revealed that OGD/RP increased the number of apoptotic cells and nuclear condensation, while pretreatment with different concentrations of artemisinin for 2 hours markedly reduced the number of apoptotic cells and nuclear condensation caused by OGD/RP (Fig. 1E-F). Flow cytometry analysis with Annexin V-FITC/PI staining revealed that the model group (OGD/RP) had a significantly higher rate of apoptosis than the control group and that pretreatment with 25 μM artemisinin significantly protected the cells against OGD/RP-induced apoptosis significantly reducing the rate of apoptotic cells (Fig. 1G-H). Consistent with these findings, caspase 3 activity measurements indicated that 2 hours of artemisinin (25 μM) pretreatment attenuated OGD/RP induced activation of caspase 3 (Fig. 1I-J).

### 3.3 Artemisinin attenuated OGD/RP-induced reactive oxygen species (ROS) production and loss of mitochondrial membrane potential (Δψm) in PC12 cells

**Figure 2:**
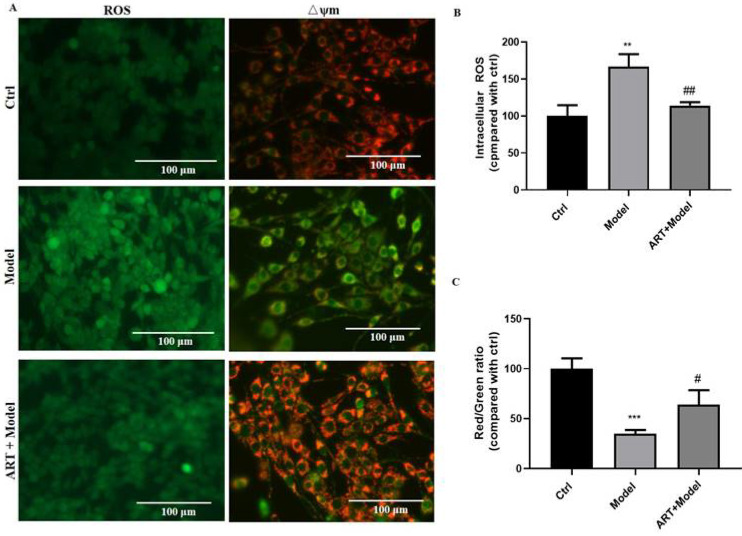
** Artemisinin attenuated OGD/RP-induced reactive oxygen species (ROS) production and loss of mitochondrial membrane potential (Δψm) in PC12 cells.** (A) Cells were pretreated with 25 μM artemisinin and then subjected to OGD/RP. Representative images of ROS levels and mitochondrial membrane potential. Scale bar: 100 μm. (B) Quantitative analysis of ROS levels. (C) Quantitative analysis of mitochondrial membrane potential. ^**^ *p* < 0.05, ^***^ *p* < 0.001, versus ctrl group; ^#^* p* < 0.05, ^##^* p* < 0.01, versus model group were considered significantly different. The assay was repeated at least 3 times.

To further understand the mechanisms underlying in the protective effect of artemisinin, ROS production and mitochondrial membrane potential were measured. As shown in Fig. 2, OGD/RP resulted in a remarked increase in intracellular ROS levels and a decrease in mitochondrial membrane potential (mitochondrial damage), whereas pretreatment with 25 μM artemisinin significantly inhibited OGD/RP-induced excessive ROS production and the loss of Δψm.

### 3.4 The ERK1/2/CREB/BCL2 signaling pathway mediated the protective effects of artemisinin against OGD/RP-induced injury in PC12 cells

**Figure 3:**
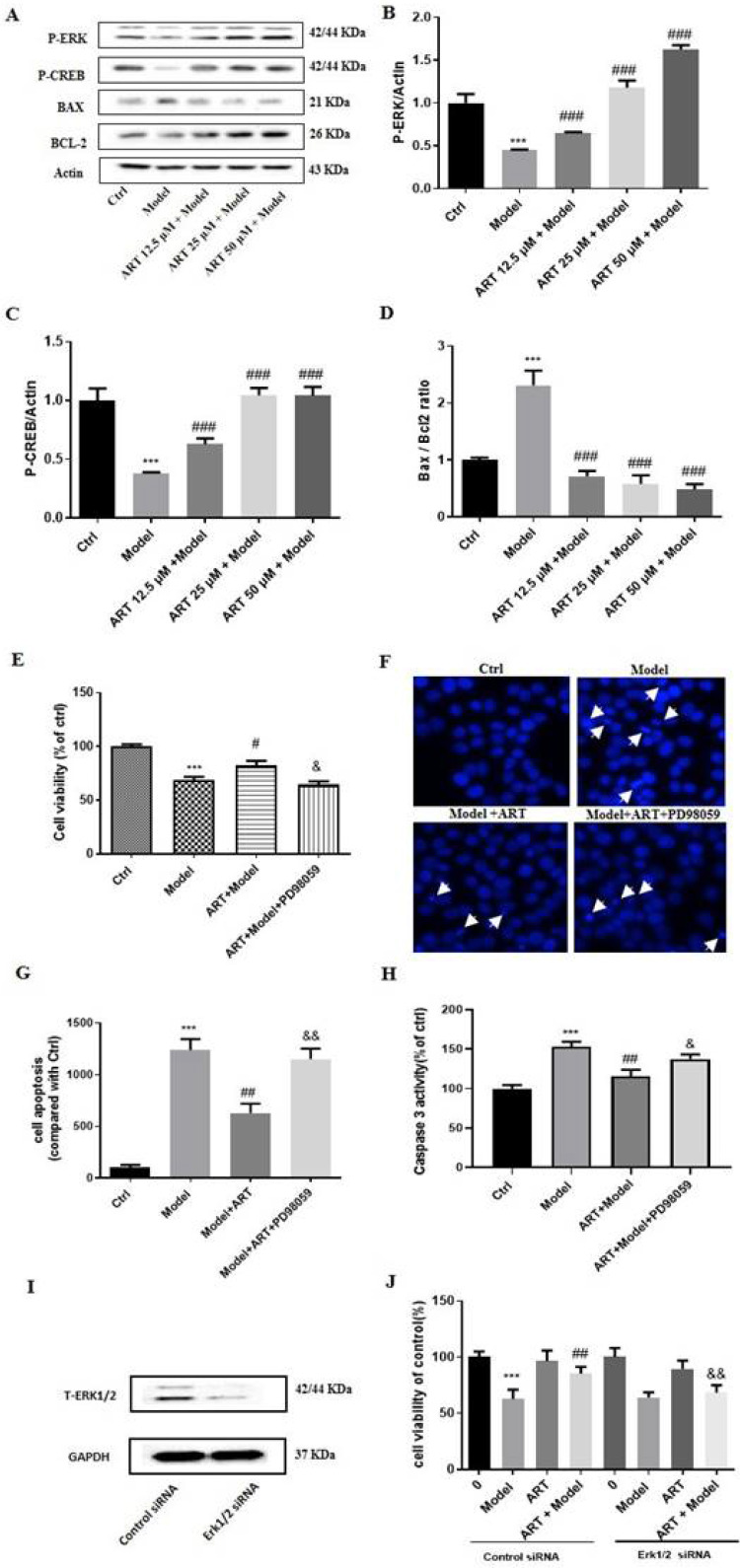
** The ERK1/2/CREB/BCL2 signaling pathway mediated the protective effects of artemisinin and ERK1/2 inhibitor PD98059 or silencing of ERK1/2 blocked the protective effect of artemisinin in PC12 cells. (A)** PC12 cells were incubated with artemisinin at the indicated concentrations and then subjected to OGD/RP conditions. The expression of phosphorylated ERK, CREB, BAX and BCL2 were detected by Western blotting. **(B-D)** Densitometric analysis of the immunoblots expressed as percentage of control. Moreover, PC12 cells were pre-treated with 25 μM PD98059 for 40 min, then treated with 25 μM artemisinin for 2 hours and then induced with OGD for 4 hours followed by 20 hours of reperfusion. Cell viability was determined by MTT assay** (E)**, cell apoptosis was determined by Hoechst 33342 staining **(F, G)** and caspase 3 activity **(H)**. **(I)** Cells were transfected with ERK1/2 siRNA and the expression of ERK was detected by Western blotting. GAPDH were used as a loading control.** (J)** Cells were transfected with ERK1/2 siRNA, further treated with 25 μM artemisinin and then induced with OGD for 4 hours followed by 20 hours of reperfusion. Cell viability was measured by MTT assay. ^***^ *p* < 0.001, versus ctrl group; ^#^* p* < 0.05, ^##^* p* < 0.01,^###^* p* < 0.001, versus model group; ^&^* p* < 0.05, ^&&^* p* < 0.01,versus ART+ model group were considered significantly different. The assay was repeated at least 3 times.

The pathways involved in the artemisinin-mediated neuroprotective effect were examined by western blotting and revealed that the phosphorylation levels of ERK1/2 and CREB gradually increased after artemisinin administration, indicating the involvement of this pathway (Fig. 3A-C). Moreover, OGD/RP induced a significant increase in the BAX/BCL2 ratio, and this effect was reversed by treatment (Fig. 3D). To gain further insight into the role of ERK1/2 in the protective effect of artemisinin, we used PD98059 (ERK1/2 inhibitor) and siRNA specific for ERK1 and ERK2 to knock down its expression in PC12 cells. As expected, preconditioning the cells with 25 μM PD98059 or downregulation ERK1/2 notably blocked the protective effect of artemisinin, including the increase in viability (Fig. 3 E and J) and decrease in apoptosis (Fig. 3 F-H).

### 3.5 Artemisinin protected primary cultured neurons against OGD/RP-induced injury via ERK1/2

**Figure 4:**
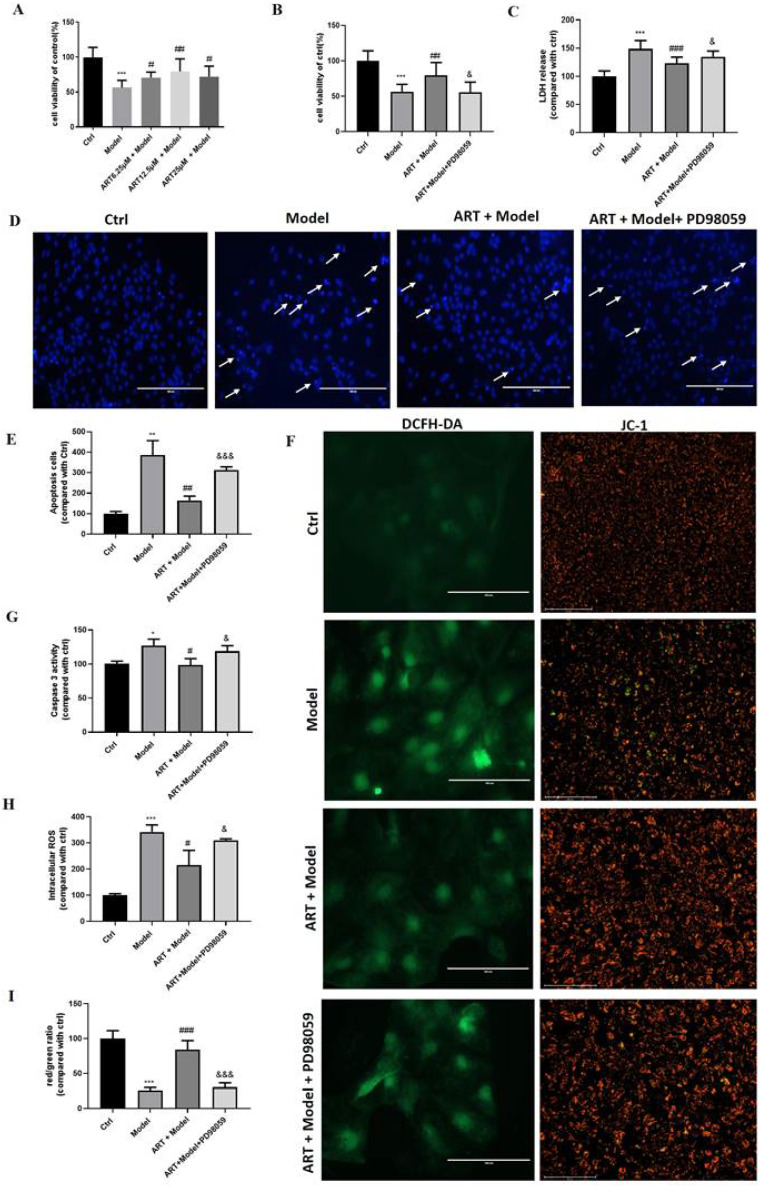
** Artemisinin protected primary cultured neurons against OGD/RP induced injury via the ERK1/2.** (A) Primary cultured neurons were pre-treated with different concentrations of artemisinin for 2 h and then induced with OGD for 4 hours followed by 20 hours of reperfusion. Cell viability was determined by MTT assay. Primary cultured neurons pretreated with 20 μM PD98059 for 30 min were treated with 12.5 μM artemisinin for 2 hours and then induced with OGD for 4 hours followed by 20 hours of reperfusion. Cell viability was determined by MTT assay (B), LDH release was measured by LDH assay (C), Cell apoptosis detected by Hoechst 33342 staining (D, E) and Caspase 3 activity (G). Scale bar: 100 μm. Reactive oxygen species (ROS) production and mitochondrial membrane potential (Δψm) were measured by DCFH-DA and JC-1 assays (F, H, I). Scale bar: 100 μm and 150μm. ^*^ *p* < 0.05, ^**^ *p* < 0.01, ^***^ *p* < 0.001, versus ctrl group; ^#^* p* < 0.05, ^##^* p* < 0.01, ^###^* p* < 0.001, versus model group; ^&^* p* < 0.05, ^&&&^* p* < 0.001, versus ART+ model group were considered significantly different. The assay was repeated at least 3 times.

To verify whether the neuroprotective effects of artemisinin against OGD/RP insult were not specific to PC12 cells, we also examined its protective effects on primary cultured neuronal cells. As expected, artemisinin also protected primary cultured neurons against OGD/RP-induced injury in a concentration-dependent manner. As shown in Fig. 4A, the protective effect of artemisinin was significant at concentration of 6.25 μM, peaked at 12.5 μM, and slightly decreased at 25 μM. Moreover, the protective effect of artemisinin was inhibited by PD98059 treatment of primary cultured neurons (Fig. 4 B-J). These results were consistent with the results obtained in PC12 cells, further confirming the involvement of the ERK1/2 pathway in the neuroprotective effect of artemisinin.

### 3.6 Artemisinin decreased ischemia/reperfusion-induced cerebral infarction and brain water content

**Figure 5:**
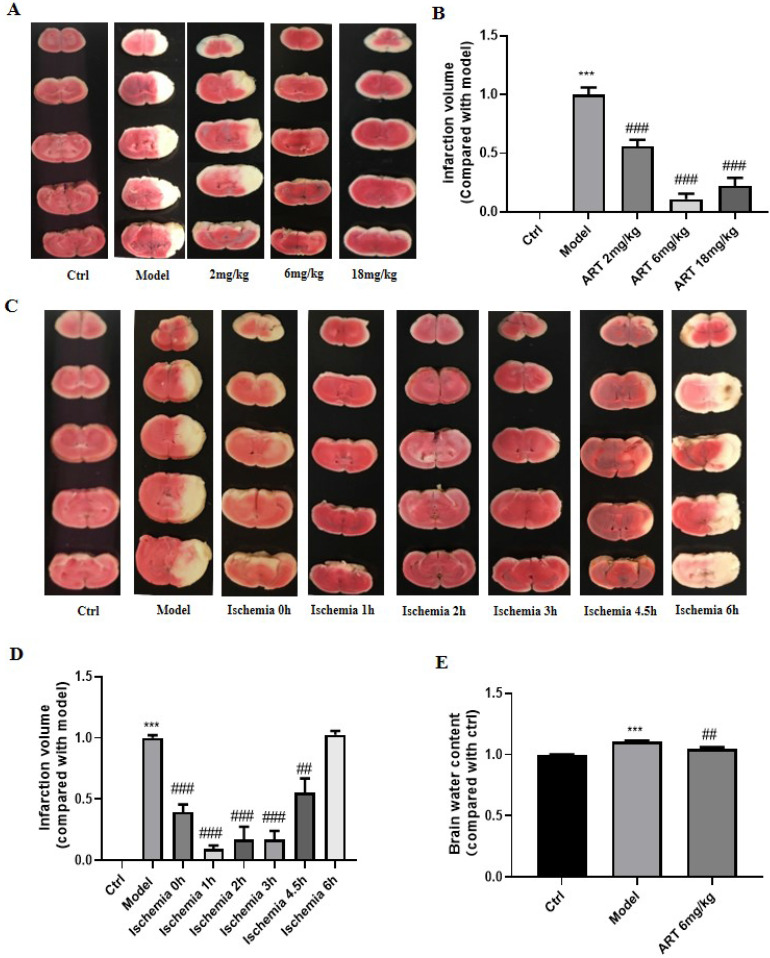
** Artemisinin decreased ischemia/reperfusion-induced cerebral infarction and brain water content** (A) Brain infarct volume was measured 24 hours after MCAO treatment with different doses of artemisinin (2, 6, 18 mg/kg) and the representative pictures of the TTC staining were shown. (B) Quantification of the infarction volume of A. (C) Brain infarct volume was measured at different time points of artemisinin treatment after ischemia (eg. Ischemia 0 h means artemisinin treatment 1 hour before reperfusion; Ischemia 1 h means artemisinin treatment while reperfusion; Ischemia 2 h means artemisinin treatment 1 h after reperfusion.) (D) Quantification of the infarction volume of C. (E) The brain water content was measured 24 hours after MCAO treatment with artemisinin (6 mg/kg).^ ***^ *p* < 0.001, versus ctrl group; ^##^* p* < 0.01, ^###^* p* < 0.001, versus model group were considered significantly different. The assay was repeated at least 3 times.

To evaluate the protective effect of artemisinin *in vivo*, a middle cerebral artery occlusion (MCAO) animal model was established, and the effect of artemisinin on infarction size was measured by TTC staining. MCAO mice showed increased infarct volumes, which were significantly decreased in the artemisinin-treated group in a time- and dose- dependent manner (Fig. 5A-D). Moreover, treatment with 6mg/kg artemisinin significantly decreased the brain water content in the ischemic hemisphere compared with that in the model group (Fig. 5E). The neuroprotective effect of artemisinin was examined in MCAO rats, and treatment decrease the infarction volume and brain water content (Fig. S2 A-C).

### 3.7 Artemisinin improved the recovery of motor function and decreased neuronal injury induced by ischemia/reperfusion

**Figure 6:**
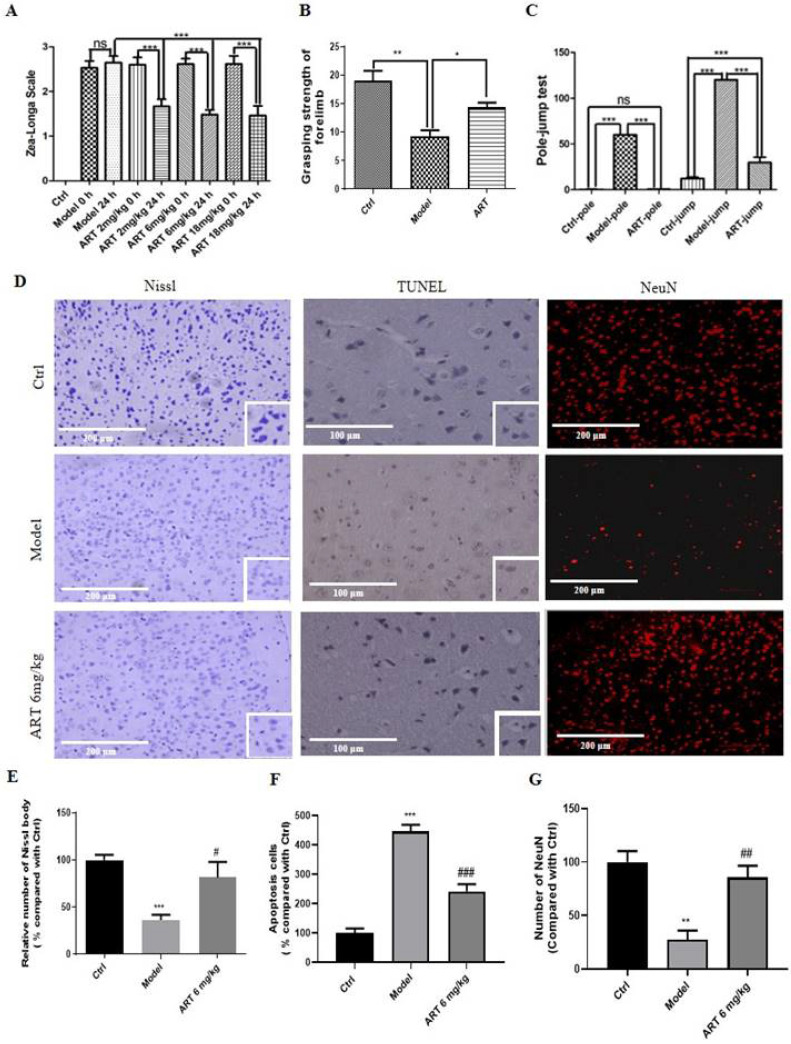
** Artemisinin improved the recovery of motor function and decreased neuronal injury induced by ischemia/reperfusion.** (A) Evaluation of the effect of different doses of artemisinin (2, 6, 18 mg/kg) in the MCAO mice neurological deficits using Zea-Longa Scale neurological score. (B) Behavioral evaluation of the grasping strength of the forelimb with 6 mg/kg artemisinin. (C) Behavioral evaluation of the pole-jump test with 6 mg/kg artemisinin. ^*^ p < 0.05,^ **^ *p* < 0.01, ^***^ *p* < 0.001 were considered significantly different. (D) Representative images of Nissl staining, TUNEL staining and immunofluorescence of NeuN from the different groups. Scale bar: 100 μm and 200 μm. (E) Quantitative analysis of Nissl staining. (F) Quantitative analysis of TUNEL staining. (G) Quantitative analysis of immunofluorescence of NeuN.^ **^ *p* < 0.01, ^***^ *p* < 0.001, versus ctrl group;^ #^ *p* < 0.05, ^##^ *p* < 0.01,^ ###^ *p* < 0.001 versus model group. The assay was repeated at least 3 times.

The effect of artemisinin on the neurological deficits, grip strength and motor behaviours of the MCAO mouse model was measured using different behaviour tests. As shown in Fig. 6A, before artemisinin administration, the animals in the model group and model plus artemisinin treatment groups exhibited significantly increased neurological deficits relative to animals in the ctrl group. After 24 hours of artemisinin treatment, the mice showed significantly lower neurological deficits than untreated animals in the model group. Similar results were obtained in the MCAO rat model (Fig. S2D). Moreover, artemisinin promoted significant recovery of grip strength (Fig. 6B) and improved of the pole jump performance compared with those in the model group (Fig. 6C).

Histopathological changes were analyzed by Nissl staining (Fig. 6D-Nissl, E) and revealed that Nissl bodies were numerous in the ctrl group, indicating that the cell condition was improved; in the model group, the number of Nissl bodies was reduced, and this pathological change was attenuated by 6 mg/kg artemisinin. The effect of artemisinin on apoptosis was analyzed by TUNEL staining (Fig. 6D-TUNEL, F), and the number of apoptotic cells was significantly increased in the model group, while artemisinin reversed the effect of ischemic injury, significantly reducing the number of apoptotic cells. In addition, artemisinin reversed the decrease in the number of neurons observed in the model group (Fig. 6D-NeuN, G).

### 3.8 The ERK1/2/CREB/BCL2 signaling pathway mediated the protective effects of artemisinin in an ischemia reperfusion mouse model

**Figure 7:**
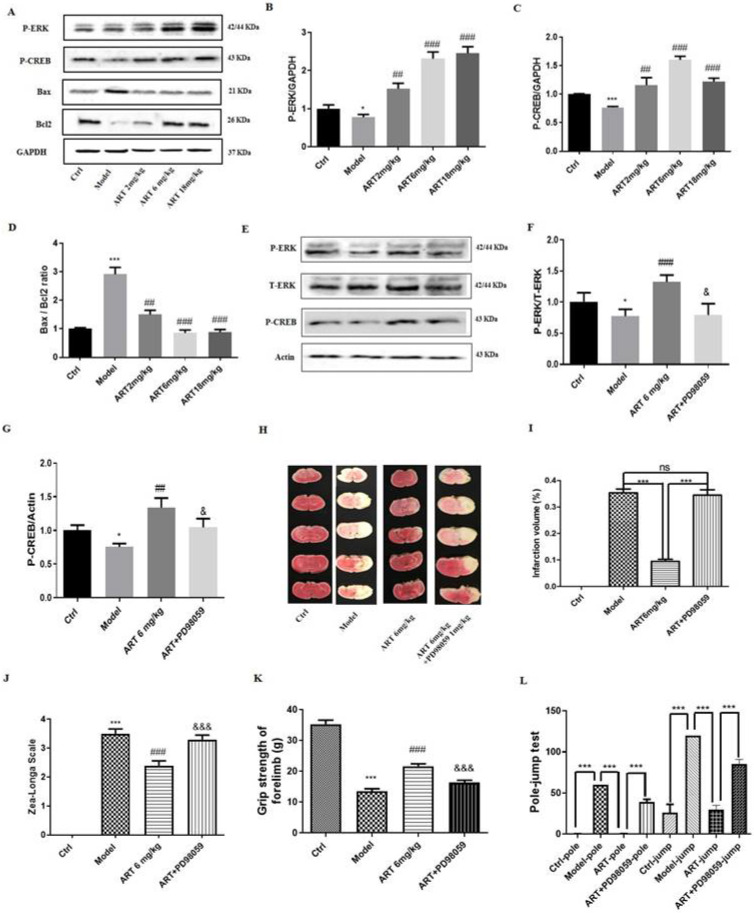
** The ERK1/2/CREB/BCL2 signaling pathway mediated the protective effects of artemisinin in an ischemia reperfusion mouse model. (A)** Western blot analysis of P-ERK, P-CREB, BAX and BCL2 protein levels 24 hours after MCAO treatment with different doses of artemisinin (2, 6, 18 mg/kg) and normalized with GAPDH. **(B)** Quantification of the P-ERK protein expression levels. **(C)** Quantification of the P-CREB protein expression levels. **(D)** Quantification of the BAX/BCL2 protein expression ratio. **(E-G)** Western blot analysis and quantification of P-ERK and P-CREB protein levels 24 hours after MCAO treatment with 1 mg/kg PD98059 followed by 6 mg/kg artemisinin and normalized with actin. **(H)** The brain infarct volume was measured 24 hours after MCAO treatment with 1 mg/kg PD98059 followed by 6 mg/kg artemisinin. Representative images of the TTC staining. **(I)** Quantification of the infarction volume of H.** (J)** Evaluation of the neurological deficits using Zea-Longa Scale neurological score of each group. **(K)** Behavioral evaluation of the grasping strength of the forelimb. **(L)** Behavioral evaluation of the pole-jump test. ^*^ *p* < 0.05, ^***^ *p* < 0.001, versus ctrl group; ^##^* p* < 0.01, ^###^* p* < 0.001, versus model group; ^&^* p* < 0.05, ^&&&^* p* < 0.001 versus ART + model group were considered significantly different. The assay was repeated at least 3 times.

As the results of the *in vitro* experiments suggested the involvement of the ERK/CREB/BCL2 signaling pathway in the protective effect of artemisinin, we examined the involvement of this pathway *in vivo.* As shown in Fig. 7A-D, the expression levels of P-ERK1/2 and P-CREB were significantly increased, and the ratio of BAX/BCL2 was significantly decreased in the artemisinin treated group compared with the model group. Further inhibition of ERK1/2 with the ERK1/2 inhibitor PD98059 (1mg/kg) prevented the protective effects of artemisinin (Fig. 7E-I). Moreover, after PD98059 administration, the improvements in neurological deficits, grip strength and pole jump performance induced by artemisinin were reversed (Fig. J-L). These results, which were consistent with those obtained *in vitro*, indicate that artemisinin attenuated ischemic stroke-induced apoptosis through activation of the ERK1/2/CREB/BCL-2 signaling pathway. In addition, we examined the levels of superoxide dismutase (SOD) and malondialdehyde (MDA) *in vitro* and* in vivo*, which are widely used to evaluate the effect of oxidative stress 35. The results showed that SOD levels were decreased and MDA levels were increased in the model group, and artemisinin reversed the effect of ischemia injury on SOD and MDA levels (Fig. S3A-D). Further investigation of the role of astrocytes, revealed that animals in the model group exhibited astrocytic activation in the brain, and this effect was inhibited by artemisinin treatment (Fig. S3E-F). Furthermore, artemisinin decreased the levels of the inflammatory cytokines IL-18, IL-1β and NF-κB after MCAO injury (Fig. S3G-J). These results suggest that artemisinin may have antioxidative and anti-inflammatory effects.

## 4. Discussion

Cerebral ischemic and reperfusion injury include a transient or permanent blockage of blood flow, that results in cerebral infarction, ultimately leading to disability or death 36. Currently, there are no effective treatments for stroke, with the exception of antithrombolytic drugs and hypothermia strategies, demonstrating the necessity of safe and more effective drugs 37. In this study, we found that artemisinin could protect neuronal cells from ischemia stroke injury in both cellular and animal experimental models and that this neuroprotective effect was mediated by the ERK/CREB/BCL2 signaling pathway.

Artemisinin has been used as a safe and affordable antimalarial drug for decades. Because it is able to cross the blood brain barrier (BBB), artemisinin was recently reported to also have a neuroprotective effect on PC12 cells 5, 38, human retinal pigment epithelial cells 29, 39, SH-SY5Y cells 7, rat bone marrow-derived mesenchymal stem cells 8, ventral spinal cord 4.1 (VSC4.1) cells 40 and in an AD mouse model 34 and brachial plexus injury mouse model 40. However, there have been no studies reporting the neuroprotective effect of artemisinin in the treatment of ischemic stroke. Thus, our study was the first time to evaluate the neuroprotective effect of artemisinin and the related signaling pathways in ischemic stroke using an OGD/RP cellular model and an MCAO animal model. The obtained results provide evidence of a new therapeutic candidate for the treatment of ischemic stroke.

Apoptosis leads to the fragmentation of DNA, degradation of cytoskeletal and nuclear proteins and the formation of apoptotic bodies 41. Ischemic stroke is characterized by pivotal molecular events that initiate apoptosis, such as excitotoxicity, calcium overload and free radical overproduction 22. Previous reports have suggested that apoptosis may contribute to a significant proportion of neuronal death following acute brain ischemia 42, 43, and apoptosis within the ischemic penumbra may occur after several hours or even several days. Caspase-3 plays an important role in a variety of apoptotic signaling pathways and may also be involved in ischemic stroke 44, 45. Hence, in the present study, apoptosis was examined both *in vitro* and *in vivo*. Artemisinin increased cell viability, decreased LDH activity, reduced apoptosis and attenuated the production of ROS and the loss of mitochondrial membrane potential in the OGD/RP cellular model. Additionally, artemisinin inhibited the ratio of the apoptosis-related protein BAX/BCL2 and activated signaling pathways related to cell survival. Further validation of the neuroprotective effect of artemisinin in an MCAO animal model revealed that this treatment could attenuate the infarction volume and the brain water content, improve neurological deficits, restore grip strength and recover motor function. Moreover, artemisinin significantly inhibited apoptosis, oxidative stress and neuroinflammation, suggesting that the neuroprotective effect of artemisinin on ischemic stroke may occur through antiapoptotic, antioxidative and anti-inflammatory effects.

ERK1/2 regulates several cellular processes such as proliferation, differentiation, and survival 46. It has been shown that neuroprotection in ischemic stroke could be regulated by ERK 47. These findings of this study also suggest that the protective effect of artemisinin may be mediated by ERK signaling pathway activation, since the phosphorylation of ERK was increased by artemisinin treatment. Moreover, cell survival can be further regulated by the activity of the transcription factor CREB (downstream of ERK) 48, 49 and by BAX and BCL2 50. In this study, artemisinin reversed the ischemic stroke-induced decrease in the phosphorylation of ERK and CREB and increase in the ratio of BAX/BCL2. Furthermore, specific inhibition of ERK1/2 or ERK1/2 silencing reversed the neuroprotective effect of artemisinin *in vitro* and *in vivo*. Collectively, these results provide mechanistic evidence that the ERK1/2/CREB/BCL2 signaling pathway plays a role in the neuroprotective effect of artemisinin on ischemic stroke.

## 5. Conclusion

**Figure 8:**
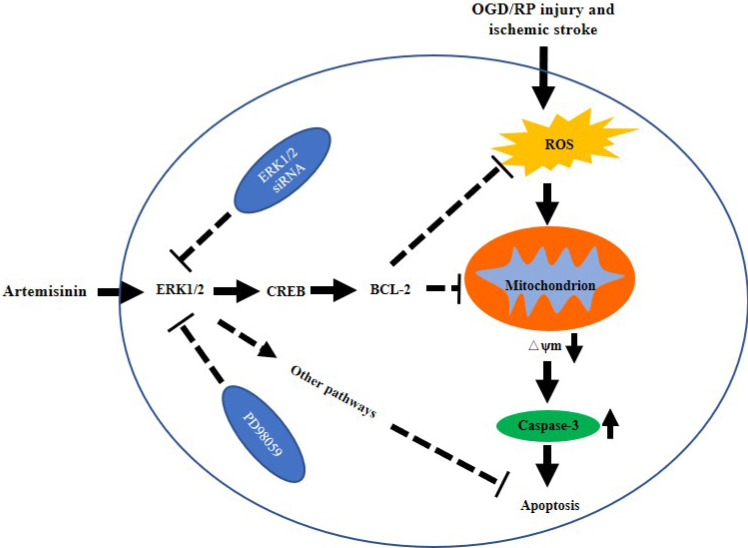
** The possible mechanism of artemisinin mediated neuroprotection against ischemic stroke-induced injury.** Artemisinin stimulated ERK1/2 related signaling pathway in PC12 cells and the brain of MCAO mice model, resulting in the reduction of oxidative stress and inhibition of apoptosis and neuroinflammation.

In conclusion, we showed that artemisinin has protective effects on PC12 cells, primary cultured neurons and an MCAO animal model, and demonstrated that artemisinin could attenuate ischemic stroke induced apoptosis through activation of the ERK1/2/CREB/BCL-2 signaling pathway *in vitro* and *in vivo* (Fig. 8). Our findings reveal the molecular mechanisms underlying the antiapoptotic effect of artemisinin on ischemic stroke models, supporting its potential use as a novel neuroprotective agent to prevent and treat ischemic stroke.

## Supplementary Material

Supplementary figures.
