# Supplementary material for: Artemisinin attenuated ischemic stroke induced cell apoptosis through activation of ERK1/2/CREB/BCL-2 signaling pathway in vitro and in vivo
Source: Int J Biol Sci. 2022 Jul 11;18(11):4578–94. doi: 10.7150/ijbs.69892 (PMC9295073; doi:10.7150/ijbs.69892)
Supplement: Supplementary file 1 — Supplementary figures. [file ijbsv18p4578s1.pdf]

Figure S1

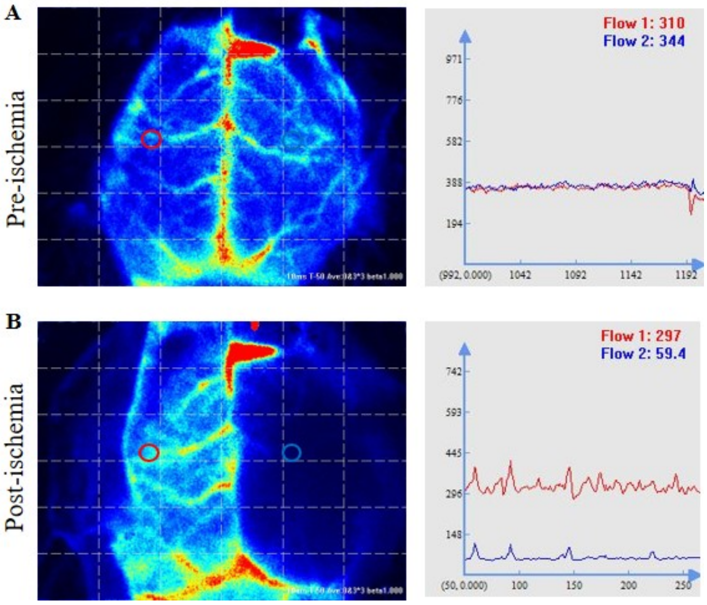

Fig.S1 Cerebral blood flow (CBF) was measured in the area of the MCA by a laser doppler flowmetry of MCAO mice model. Mice were subjected to ischemia for 1 hour and reperfusion for 24 hours. Cerebral blood flow (CBF) was measured pre- ischemia (A) and post-ischemia(B). After the model was established, the blood flow on the ischemic side (blue line) decreased when compared with the non-ischemic side (red line).

Figure S2

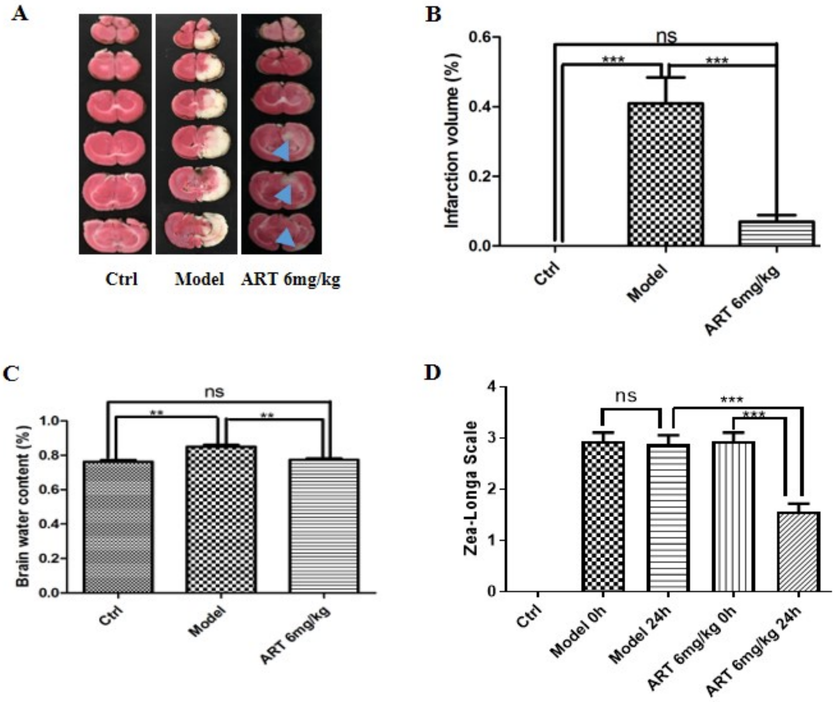

Fig.S2 Artemisinin decreased the ischemia reperfusion induced cerebral infarction, the brain water content and improved the neurological deficits of MCAO rat model. MCAO model was also established in Sprague Dawley (SD) rats using the same method. (A) Representative images of the TTC staining of brain sections from MCAO rats model treated with 6mg/kg artemisinin. (B) Quantification of the infarction volume of A. (C) Brain water content of MCAO rats model treated with 6 mg/kg artemisinin. (D) Evaluation of the neurological deficits of MCAO rats treated with 6 mg/kg artemisinin. ** $p < 0.01$, *** $p < 0.001$ were considered significantly different. The assay was repeated at least 3 times.

Figure S3

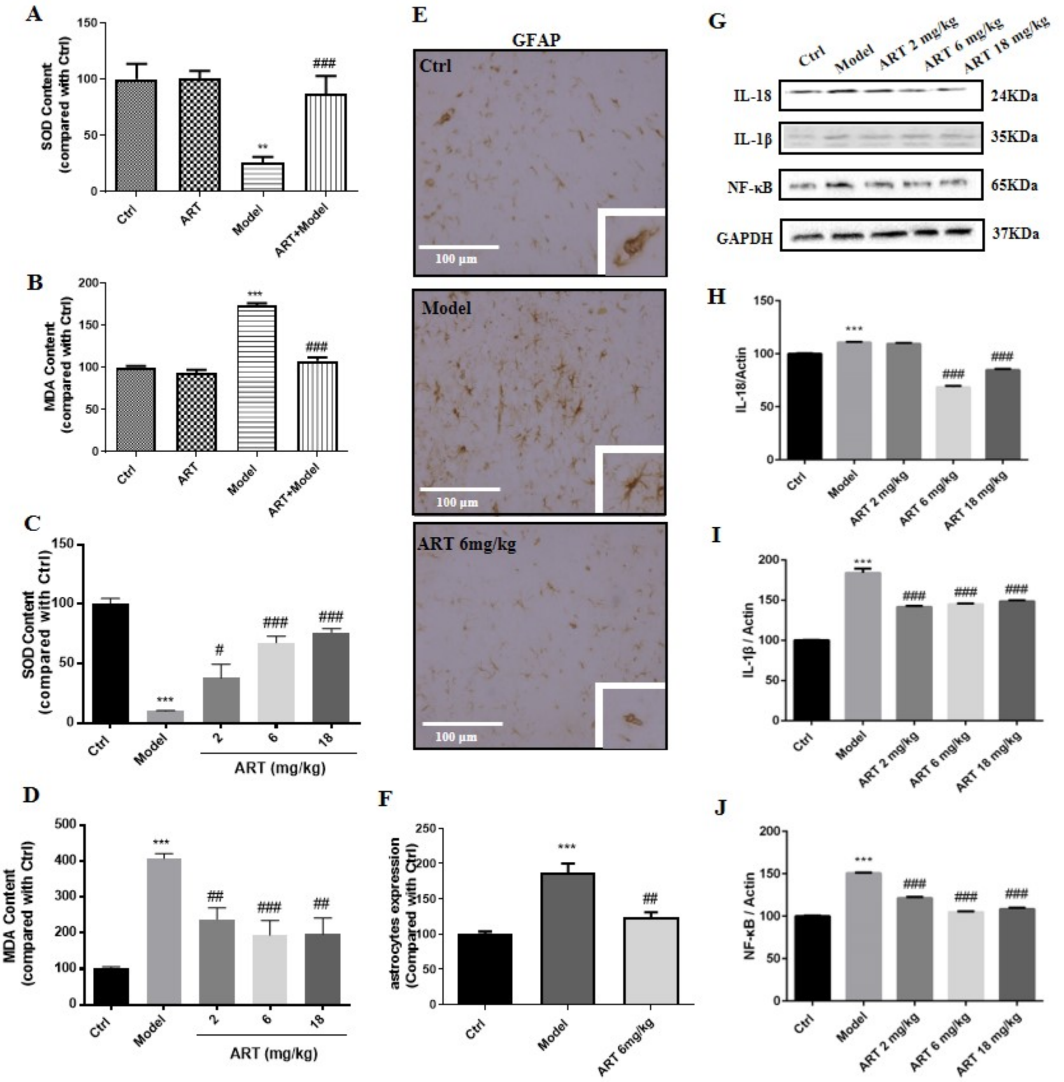

Fig.S3 Artemisinin suppressed ischemia reperfusion induced oxidative stress and inflammation. (A, B) PC12 cells were treated with 25 μM artemisinin for 2 hours and then exposed to OGD for 4 hours followed by 20 hours of reperfusion. The contents of SOD and MDA were then measured. (C, D) SOD and MDA activities were further detected 24 hours after MCAO treatment with 2, 6 and 18 mg/kg artemisinin (E) Representative images of the brain cortex of mice of the different groups, stained immunocytochemically for GFAP. Scale bar: 100 μm. (F) GFAP quantification. (G) The expression of IL-18, IL-1β, and NF-κB was detected by western blotting and normalized with GAPDH . (H-J) Quantification of the representative protein bands from western blotting of G. ** $p < 0.01$, *** $p < 0.001$, versus ctrl group; $^{\#}$ $p < 0.05$, $^{\#\#}$ $p < 0.01$, $^{\#\#\#}$ $p < 0.001$ versus model group. The assay was repeated at least 3 times.
